# Longitudinal Assessment of Third-Trimester Fetal Biometry and Cerebroplacental Ratio for Predicting Adverse Perinatal Outcomes in an Unselected Obstetric Population: A Prospective Cohort Study

**DOI:** 10.3390/jcm15166316

**Published:** 2026-08-15

**Authors:** Emine Merve Turhan, Mustafa Koçar, Cenk Soysal, Yasemin Taşcı

**Affiliations:** 1 Department of Obstetrics and Gynecology, Kütahya Health Sciences University, Kütahya 43100, Türkiye; eminemerve.turhan@ogr.ksbu.edu.tr (E.M.T.); cenk.soysal@ksbu.edu.tr (C.S.); yasemin.tasci@ksbu.edu.tr (Y.T.); 2 Department of Obstetrics and Gynecology, Kütahya City Hospital, Kütahya 43100, Türkiye

**Keywords:** cerebroplacental ratio, fetal biometry, Doppler ultrasonography, estimated fetal weight, adverse perinatal outcome, low-risk pregnancy, neonatal outcome

## Abstract

**Background**: The clinical value of the cerebroplacental ratio (CPR) for predicting adverse perinatal outcomes in unselected obstetric populations remains uncertain. We aimed to evaluate the prognostic performance of serial third-trimester fetal biometry and Doppler-derived CPR and to compare their ability to identify adverse perinatal outcomes and neonatal growth abnormalities. **Methods**: In this prospective longitudinal cohort study, 100 consecutive pregnancies from an unselected obstetric population underwent standardized ultrasonographic examinations at both 28 and 37 weeks of gestation. Fetal biometric measurements, estimated fetal weight (EFW), amniotic fluid index, umbilical and middle cerebral artery Doppler indices, and CPR were recorded. Maternal, obstetric, delivery, and neonatal data were collected prospectively. Receiver operating characteristic (ROC) curve analyses and parsimonious multivariable regression models, accompanied by 1000-sample bootstrap internal validation, were performed to evaluate the independent predictive capacity of fetal biometry and CPR metrics. **Results**: Gestational age-specific CPR percentiles were associated with expected physiological Doppler changes but showed limited associations with obstetric and neonatal outcomes. In separate parsimonious regression models optimized for event-per-variable ratios, neither 28-week nor 37-week CPR independently predicted composite adverse perinatal outcomes, NICU admission, low Apgar scores, or abnormal umbilical cord blood pH. Rigorous bootstrap internal validation confirmed that optimism-corrected area under the curve (AUC) values for CPR models remained close to chance (range: 0.463–0.507). In contrast, 37-week EFW independently predicted neonatal birth weight, while both 37-week EFW and EFW percentile demonstrated good discriminatory performance for identifying small-for-gestational-age neonates (AUC = 0.812 and 0.833, respectively; both *p* < 0.001). Maternal body mass index and late-pregnancy fetal biometry also showed moderate discriminatory performance for predicting large-for-gestational-age neonates. Overall, late-pregnancy biometry outperformed CPR for identifying growth abnormalities, whereas neither modality alone accurately predicted composite adverse perinatal outcomes. **Conclusions**: In an unselected obstetric population, serial third-trimester CPR provided limited additional prognostic information beyond routine fetal biometry. Late-pregnancy fetal biometry, particularly 37-week estimated fetal weight, demonstrated superior performance for identifying neonatal growth abnormalities. These findings support the continued use of conventional fetal biometry as the primary component of routine third-trimester surveillance, with CPR serving as a complementary rather than standalone Doppler parameter.

## 1. Introduction

Pregnancy requires complex physiological interactions among the maternal, placental, and fetal systems. Normal fetal development depends on adequate uteroplacental perfusion. Impairments in these adaptive mechanisms can compromise nutrient delivery, potentially leading to fetal growth restriction (FGR), preterm birth, or adverse perinatal outcomes. Accurate assessment of fetal well-being remains a cornerstone of modern obstetric care [1,2,3].

Ultrasonographic fetal biometry is essential for monitoring fetal growth throughout pregnancy. Parameters such as abdominal circumference (AC) and estimated fetal weight (EFW) routinely identify deviations from expected growth trajectories and detect fetuses at risk for growth restriction [4,5]. However, these biometric parameters primarily reflect fetal size rather than functional circulatory adaptation. Consequently, reliance on fetal biometry alone may fail to identify pregnancies at risk for adverse perinatal outcomes [6].

Doppler ultrasonography provides a non-invasive assessment of uteroplacental and fetoplacental circulation. Doppler evaluation of the umbilical artery (UA) and middle cerebral artery (MCA) detects fetal cardiovascular adaptations to placental insufficiency and hypoxia [7]. A key compensatory response is the redistribution of cardiac output toward vital organs, known as the brain-sparing effect. The cerebroplacental ratio (CPR), calculated as MCA PI/UA PI, serves as a sensitive marker for this compensatory response. While CPR is associated with adverse obstetric and neonatal outcomes in several studies, reported findings remain inconsistent across different screening populations and clinical settings [8,9].

The prognostic performance of biometric and Doppler metrics evolves throughout pregnancy, as both fetal growth velocity and circulatory adaptation change with gestational age [10]. Assessments performed at approximately 28 weeks capture early growth trajectories, whereas term evaluations near 37 weeks reflect cumulative placental capacity immediately before delivery [11]. Most previous studies evaluated these parameters cross-sectionally or at a single time point. The incremental value of sequential biometric and Doppler assessments during routine late-pregnancy surveillance remains incompletely understood, particularly in unselected obstetric cohorts [12,13]. To our knowledge, prospective longitudinal studies evaluating serial fetal biometry together with CPR at both 28 and 37 weeks within the same unselected obstetric population remain limited.

Accordingly, this prospective longitudinal study evaluated the association between adverse pregnancy outcomes and sequential fetal assessments—including biometric parameters (biparietal diameter [BPD], head circumference [HC], abdominal circumference [AC], femur length [FL], estimated fetal weight [EFW], amniotic fluid index [AFI], and placental localization) and Doppler-derived CPR—obtained at 28 and 37 weeks of gestation. We aimed to determine whether combining longitudinal biometry with Doppler-derived CPR improves the identification of pregnancies at risk for adverse perinatal outcomes within an unselected obstetric population.

## 2. Materials and Methods

### 2.1. Study Design and Participant Selection

This prospective longitudinal observational study was conducted between December 2024 and May 2025 at the Department of Obstetrics and Gynecology, Kütahya Health Sciences University, Kütahya City Hospital, Kütahya, Türkiye. The study protocol was approved by the Non-Interventional Clinical Research Ethics Committee of Kütahya Health Sciences University (20 September 2024; Decision No. 118). Written informed consent was obtained from all participants before enrollment.

Eligible pregnant women attending routine antenatal care during the study period were consecutively recruited from the investigator’s outpatient clinic to establish a prospective, unselected obstetric population. Women were eligible if they were between 18 and 40 years of age, had a singleton pregnancy, had no chronic systemic disease requiring regular medication, attended routine antenatal visits at both 28 and 37 weeks of gestation, agreed to participate in the study, and planned to continue antenatal follow-up and deliver at Kütahya City Hospital.

Participants were prospectively followed throughout pregnancy, undergoing serial ultrasonographic and Doppler assessments at 28 and 37 weeks of gestation, and were subsequently followed until delivery for the assessment of obstetric and neonatal outcomes. Women who did not complete both the 28- and 37-week study assessments, delivered before 37 completed weeks of gestation, delivered at another institution, or withdrew from the study during follow-up were excluded from the final analysis. The final study population comprised 100 women with complete maternal, ultrasonographic, Doppler, delivery, and neonatal outcome data.

### 2.2. Clinical Assessment and Data Collection

At enrollment, maternal demographic and obstetric characteristics were obtained through participant interviews and electronic medical records. Recorded variables included maternal age, height, pre-pregnancy weight, gravidity, parity, number of previous abortions, number of living children, and previous mode of delivery. Body mass index (BMI) was calculated as pre-pregnancy weight (kg) divided by height squared (m^2^).

Gestational age was determined according to the first day of the last menstrual period and confirmed by first-trimester crown–rump length (CRL) measurements obtained during routine ultrasonography.

All participants received routine antenatal care and delivery management according to the current national guidelines of the Turkish Ministry of Health. As this was an observational study, no additional clinical interventions were performed beyond routine obstetric care. The mode of delivery (vaginal or cesarean delivery), indication for cesarean delivery, and emergency cesarean delivery performed for suspected fetal distress were prospectively recorded from the delivery records.

### 2.3. Ultrasonographic and Doppler Evaluation

All ultrasonographic and Doppler examinations were performed by a single experienced investigator (E.M.T.) using a Philips Affiniti 70 ultrasound system (Philips Healthcare, Andover, MA, USA) equipped with a 3.5–5.0 MHz convex transducer, thereby minimizing interobserver variability. All examinations were performed according to a standardized institutional protocol and were undertaken as part of routine antenatal care at 28 and 37 weeks of gestation.

Fetal biometric assessment included biparietal diameter (BPD), head circumference (HC), abdominal circumference (AC), femur length (FL), estimated fetal weight (EFW), amniotic fluid index (AFI), and placental location. For longitudinal comparison, fetal biometric parameters (BPD, HC, AC, and FL) were expressed as ultrasound-derived gestational age equivalents (days) rather than absolute biometric measurements (mm). Estimated fetal weight was calculated automatically using the Hadlock formula. For each biometric parameter, three consecutive measurements were obtained, and the mean value was used for statistical analyses.

Estimated fetal weight was categorized according to gestational age-specific percentiles as <10th percentile, 10th–90th percentile, and >90th percentile.

Doppler examinations were performed during fetal quiescence, in the absence of fetal breathing movements and uterine contractions. Umbilical artery (UA) Doppler waveforms were obtained from a free-floating segment of the umbilical cord, avoiding both the placental and fetal cord insertions. Middle cerebral artery (MCA) Doppler measurements were obtained from the proximal third of the vessel in an axial plane of the fetal head demonstrating the thalami and cavum septi pellucidi. The insonation angle was maintained below 30°, and at least three consecutive uniform waveforms were recorded before measurements were accepted.

Pulsatility index (PI) and resistance index (RI) were automatically calculated for both the UA and MCA. The cerebroplacental ratio (CPR) was subsequently calculated as the ratio of the MCA pulsatility index to the UA pulsatility index (CPR = MCA PI/UA PI).

Because CPR varies physiologically with advancing gestation, gestational age-specific 5th and 10th percentile thresholds derived from published normative reference ranges were used instead of a single fixed cut-off value. Participants were categorized according to these percentile thresholds for subsequent analyses.

### 2.4. Neonatal Assessment and Outcome Measures

Immediately after delivery, neonatal assessment was performed by the attending pediatric team. Recorded neonatal variables included birth weight, birth length, sex, 1- and 5 min Apgar scores, umbilical artery cord blood pH, and admission to the neonatal intensive care unit (NICU), including the duration of hospitalization.

Birth weight percentiles were determined according to the INTERGROWTH-21st international standards and categorized as small for gestational age (SGA; <10th percentile), appropriate for gestational age (AGA; 10th–90th percentile), and large for gestational age (LGA; >90th percentile).

The primary outcome was a composite adverse perinatal outcome, defined as the presence of at least one of the following criteria: umbilical artery cord blood pH < 7.20, a 1 min Apgar score < 7, low birth weight (<2500 g), or any neonatal intensive care unit (NICU) admission required regardless of the duration of hospitalization.

Secondary outcomes included mode of delivery, emergency cesarean delivery for suspected fetal distress, umbilical artery cord blood pH, Apgar scores, NICU admission, duration of NICU stay, birth weight, and neonatal birth weight percentile category.

### 2.5. Statistical Analysis

The distribution of continuous variables was assessed using the Kolmogorov–Smirnov test. Normally distributed continuous variables are presented as mean ± standard deviation (SD), whereas non-normally distributed variables are presented as median (interquartile range [IQR]). Categorical variables are expressed as number (percentage). For comparative analyses, participants were categorized according to gestational age-specific CPR thresholds (<5th vs. ≥5th percentile and <10th vs. ≥10th percentile) measured at both 28 and 37 weeks of gestation. Continuous variables were compared using the Independent Samples *t*-test or the Mann–Whitney U test, as appropriate. Comparisons of categorical variables were performed using the Chi-square test or Fisher’s exact test, where applicable. Receiver operating characteristic (ROC) curve analysis was performed to evaluate the discriminatory performance of maternal characteristics, fetal biometric measurements, and Doppler parameters in predicting neonatal outcomes. Discriminatory performance was expressed as the area under the curve (AUC) with corresponding 95% confidence intervals (CIs), together with optimal cut-off values, sensitivity, and specificity.

Variables included in the multivariable models were prespecified a priori on the basis of clinical relevance and the primary objectives of the study. All prespecified variables were entered simultaneously using the forced-entry (Enter) method. No automated stepwise, forward, or backward selection procedure was applied, and variable inclusion was not based on a univariable *p*-value threshold. To reduce overfitting and eliminate multicollinearity, measurements obtained at 28 and 37 weeks were evaluated in separate, parsimonious models. Highly correlated parameters, such as estimated fetal weight (EFW) in grams and EFW percentiles from the same examination, were strictly excluded from simultaneous entry.

For the linear regression models evaluating neonatal birth weight, estimated fetal weight, cerebroplacental ratio, and gestational age at birth were included due to their established clinical relationships. For the binary logistic regression models evaluating NICU admission and the composite adverse perinatal outcome, the number of predictors was restricted a priori to estimated fetal weight percentile and cerebroplacental ratio, representing fetal growth and fetal hemodynamic status, respectively. This restriction successfully optimized the events-per-variable (EPV) ratios to 8 for the NICU admission models and 13 for the composite adverse perinatal outcome models. Multicollinearity among the independent variables was assessed using variance inflation factors (VIFs) and tolerance values; VIF values below 3 and tolerance values above 0.33 indicated the absence of relevant collinearity.

Goodness of fit of the binary logistic regression models was assessed using the Hosmer–Lemeshow test with 10 risk groups. Internal validation was subsequently performed using 1000 bootstrap resamples. Optimism was estimated by comparing model performance within each bootstrap sample with performance when the bootstrap-derived model was applied to the original cohort. Optimism-corrected area under the receiver operating characteristic curve (AUC), adjusted Brier scores, and calibration slopes were calculated to ensure the structural stability of our predictive estimations. Statistical analyses were performed using IBM SPSS Statistics for Windows, Version 27.0 (IBM Corp., Armonk, NY, USA). All statistical tests were two-sided, and a *p*-value < 0.05 was considered statistically significant.

## 3. Results

The study included 100 women with complete maternal, fetal, delivery, and neonatal follow-up data. Maternal baseline characteristics are summarized in Table 1. The mean maternal age was 29.2 ± 4.7 years, the mean maternal height was 162.1 ± 6.4 cm, the mean maternal weight was 81.5 ± 16.2 kg, and the mean pre-pregnancy body mass index (BMI) was 31.1 ± 6.3 kg/m^2^. Primigravidae accounted for 37.0% of the study population, while 45.0% of women were nulliparous. A previous history of spontaneous abortion was absent in 77.0% of participants. Detailed maternal demographic, anthropometric, and obstetric characteristics are presented in Table 1.

Longitudinal fetal biometric and Doppler measurements obtained at 28 and 37 weeks of gestation are summarized in Table 2. Fetal biometric parameters, including biparietal diameter (BPD), head circumference (HC), abdominal circumference (AC), femur length (FL), and estimated fetal weight (EFW), increased between the 28- and 37-week examinations, reflecting expected fetal growth during pregnancy. Although the mean EFW percentile decreased slightly from 67.1 ± 33.0 to 61.1 ± 28.7, the majority of fetuses remained within the 10th–90th percentile range at both examinations. Doppler assessment demonstrated gestational age-related changes in fetal hemodynamic parameters. Mean cerebroplacental ratio (CPR) decreased from 2.1 ± 0.7 at 28 weeks to 1.8 ± 0.5 at 37 weeks, accompanied by corresponding changes in umbilical artery and middle cerebral artery Doppler indices. The proportion of pregnancies with CPR below the 5th percentile increased from 21.0% at 28 weeks to 32.0% at 37 weeks. Amniotic fluid volume remained within the normal range in most pregnancies at both examinations, with only a small number of cases of oligohydramnios or polyhydramnios observed during follow-up (Table 2).

Delivery characteristics and neonatal outcomes are summarized in Table 3. The mean interval between the 37-week assessment and delivery was 13.3 ± 7.1 days, with a mean gestational age at delivery of 38.6 ± 1.0 weeks. Overall, 38.0% of women had a spontaneous vaginal delivery, whereas 62.0% underwent cesarean delivery. The most common indication for cesarean section was a previous cesarean delivery (37.0%), followed by cephalopelvic disproportion (15.0%) and acute fetal distress (5.0%). Other indications, including preeclampsia, breech presentation, and fetal omphalocele, were uncommon.

The mean neonatal birth weight was 3252.5 ± 520.8 g, and the mean birth length was 49.9 ± 2.5 cm. According to the INTERGROWTH-21st standards, 12.0% of neonates were classified as small for gestational age (SGA), 72.0% as appropriate for gestational age (AGA), and 16.0% as large for gestational age (LGA). The mean umbilical artery cord blood pH was 7.3 ± 0.1. A 1 min Apgar score < 7 was observed in 6.0% of neonates. Admission to the neonatal intensive care unit (NICU) was required in 16.0% of neonates, with a mean duration of hospitalization of 0.9 ± 2.3 days. Overall, a composite adverse perinatal outcome occurred in 26.0% of pregnancies, structurally driven by 1 min Apgar scores, low birth weight, umbilical pH, and any required NICU admission trajectories (Table 3).

Comparisons of maternal characteristics, fetal growth parameters, obstetric outcomes, and neonatal outcomes according to the 28-week gestational age-specific CPR 5th percentile threshold are presented in Table 4. Maternal age, pre-pregnancy body mass index, and estimated fetal weight percentiles at both 28 and 37 weeks of gestation were comparable between pregnancies with a CPR below and above the 5th percentile. Similarly, no significant differences were observed in gestational age at delivery, the interval between the 37-week examination and delivery, or indications for cesarean delivery. Neonatal birth weight, birth weight percentile categories, neonatal sex distribution, and the frequency of the composite adverse perinatal outcome also did not differ significantly between the two groups. Although pregnancies with a 28-week CPR below the 5th percentile showed numerically higher rates of SGA neonates and lower rates of LGA neonates, these differences did not reach statistical significance (Table 4).

Comparisons according to the gestational age-specific 10th percentile CPR threshold at both 28 and 37 weeks of gestation are presented in Table 5. At 28 weeks, maternal characteristics, fetal biometric measurements, estimated fetal weight, gestational age at delivery, neonatal outcomes, and umbilical cord blood gas parameters were comparable between the two CPR groups. Pregnancies with a CPR below the 10th percentile demonstrated significantly lower MCA pulsatility and resistance indices together with higher UA pulsatility indices than those with CPR values at or above the 10th percentile (all *p* < 0.05). Similarly, at 37 weeks, maternal demographic characteristics and most fetal biometric parameters remained comparable between the groups. However, fetuses with a CPR below the 10th percentile had a significantly smaller abdominal circumference, lower absolute CPR values, higher UA pulsatility indices, and lower neonatal birth length than those with CPR values at or above the 10th percentile (all *p* < 0.05). No significant differences were observed in the remaining obstetric or adverse neonatal outcomes (Table 5).

Comparisons of maternal characteristics, fetal growth categories, obstetric outcomes, and neonatal outcomes according to the 37-week gestational age-specific CPR 10th percentile threshold are presented in Table 6.

Maternal demographic characteristics, gravidity, parity, history of abortion, mode of delivery, cesarean delivery indications, Apgar scores, umbilical cord blood pH categories, NICU admission, and the frequency of the composite adverse perinatal outcome were comparable between the two groups.

In contrast, significant differences were observed in the 37-week estimated fetal weight percentile classification. Pregnancies with a CPR below the 10th percentile showed a higher proportion of fetuses within the appropriate-for-gestational-age category and a lower proportion of large-for-gestational-age neonates compared with those with CPR values at or above the 10th percentile (*p* = 0.035).

No significant differences were observed in neonatal birth weight percentile categories or other clinically relevant perinatal outcomes between the two groups (Table 6).

Multivariable linear and binary logistic regression analyses were structurally optimized to evaluate the independent predictive capacity of longitudinal fetal biometric parameters and Doppler indices, as summarized in Table 7. Multicollinearity diagnostics for all final revised models demonstrated an excellent profile, with all variance inflation factors (VIF) remaining strictly near baseline levels (range: 1.001–1.059) and tolerance values approaching unity (range: 0.944–0.999), confirming the complete absence of collinearity biases.

In the multivariable linear regression models evaluating neonatal birth weight, both the 28-week and 37-week surveillance protocols were independently associated with birth weight outcomes. In the 28-week model, a 0.1-unit increase in the cerebroplacental ratio (CPR) was independently associated with a 15.979 g increase in birth weight (B coefficient: 15.979, 95% CI: 2.194 to 29.763, *p* = 0.024), while gestational age at birth also emerged as a significant determinant (B coefficient: 156.669 per week, 95% CI: 60.719 to 252.619, *p* = 0.002). In the 37-week model, late-pregnancy fetal size demonstrated a highly robust independent association, with a 100-g increase in EFW predicting a 71.593 g increase in actual birth weight (B coefficient: 71.593, 95% CI: 54.012 to 89.174, *p* < 0.001), alongside gestational age at birth (B coefficient: 85.185, 95% CI: 6.795 to 163.576, *p* = 0.034). Conversely, late third-trimester CPR did not independently predict neonatal birth weight (*p* = 0.250).

Parsimonious binary logistic regression models demonstrated that neither early nor late third-trimester Doppler metrics or fetal growth trajectories independently predicted adverse neonatal outcomes. For NICU admission, neither a 10-point increase in EFW percentile nor a 0.1-unit increase in CPR achieved statistical significance at 28 weeks (*p* = 0.545 and *p* = 0.473, respectively) or at 37 weeks (*p* = 0.994 and *p* = 0.383, respectively). Similarly, within the binary logistic models for the composite adverse perinatal outcome (*n* = 26 events), no independent associations were observed for EFW percentiles or CPR at either 28 weeks (*p* = 0.840 and *p* = 0.612) or 37 weeks (*p* = 0.290 and *p* = 0.865). The multivariable linear regression model originally designed for NICU length of stay was completely removed from the final analysis because 84 cases showed zero regression and only 16 neonates required admission, rendering linear parameters mathematically inappropriate.

The Hosmer–Lemeshow tests showed no evidence of structural lack of fit for the 28-week NICU model (χ^2^ = 3.661, df = 8, *p* = 0.886), the 37-week NICU model (χ^2^ = 3.314, df = 8, *p* = 0.913), the 28-week composite-outcome model (χ^2^ = 4.968, df = 8, *p* = 0.761), or the 37-week composite-outcome model (χ^2^ = 11.728, df = 8, *p* = 0.164). Internal validation via 1000 bootstrap resamples yielded optimism-corrected AUC values of 0.463 and 0.507 for the 28- and 37-week NICU models, and 0.464 and 0.485 for the corresponding composite-outcome models. These rigorous internal validation markers indicated that the apparent discriminatory performance of these parameters was highly sensitive to the unselected screening context and remained close to chance, substantiating the conclusion that third-trimester CPR metrics do not offer clinical utility as a standalone screening tool for adverse outcomes.

Receiver operating characteristic (ROC) analyses evaluating the discriminatory performance of fetal biometric measurements and Doppler indices for the composite adverse perinatal outcome are presented in Table 8. Neither fetal biometric measurements nor Doppler-derived parameters obtained at 28 or 37 weeks of gestation demonstrated significant discriminatory performance for predicting the composite adverse perinatal outcome. All area under the curve (AUC) values were close to 0.50, and none reached statistical significance (*p* > 0.05), indicating limited discriminatory performance of the evaluated individual parameters in this unselected obstetric population (Table 8).

**Figure 1:**
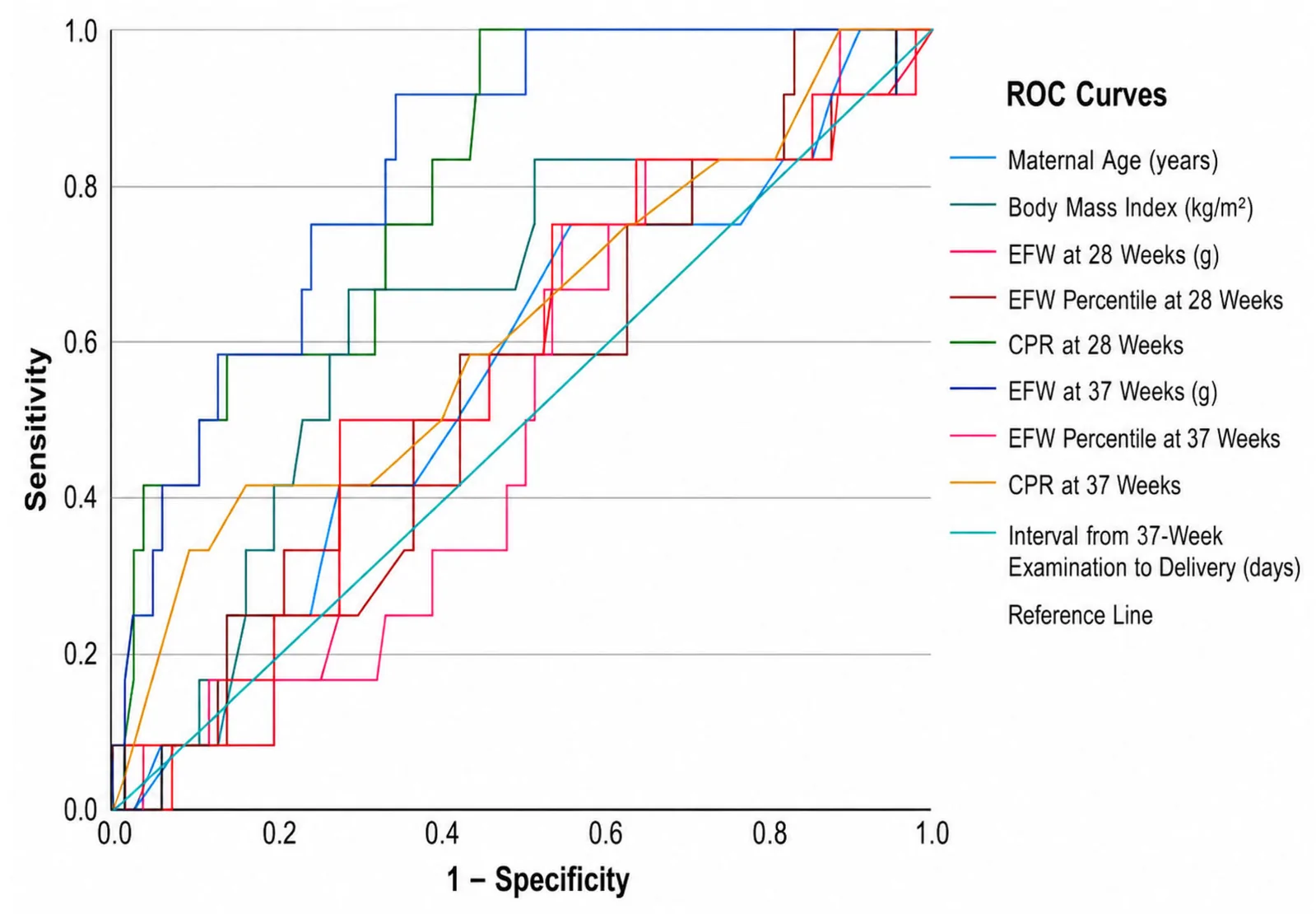
Receiver operating characteristic (ROC) curves illustrating the diagnostic performance of maternal characteristics, fetal biometric parameters, and the cerebroplacental ratio (CPR) for predicting small-for-gestational-age (SGA) neonatal outcomes within an unselected obstetric population.

**Figure 2:**
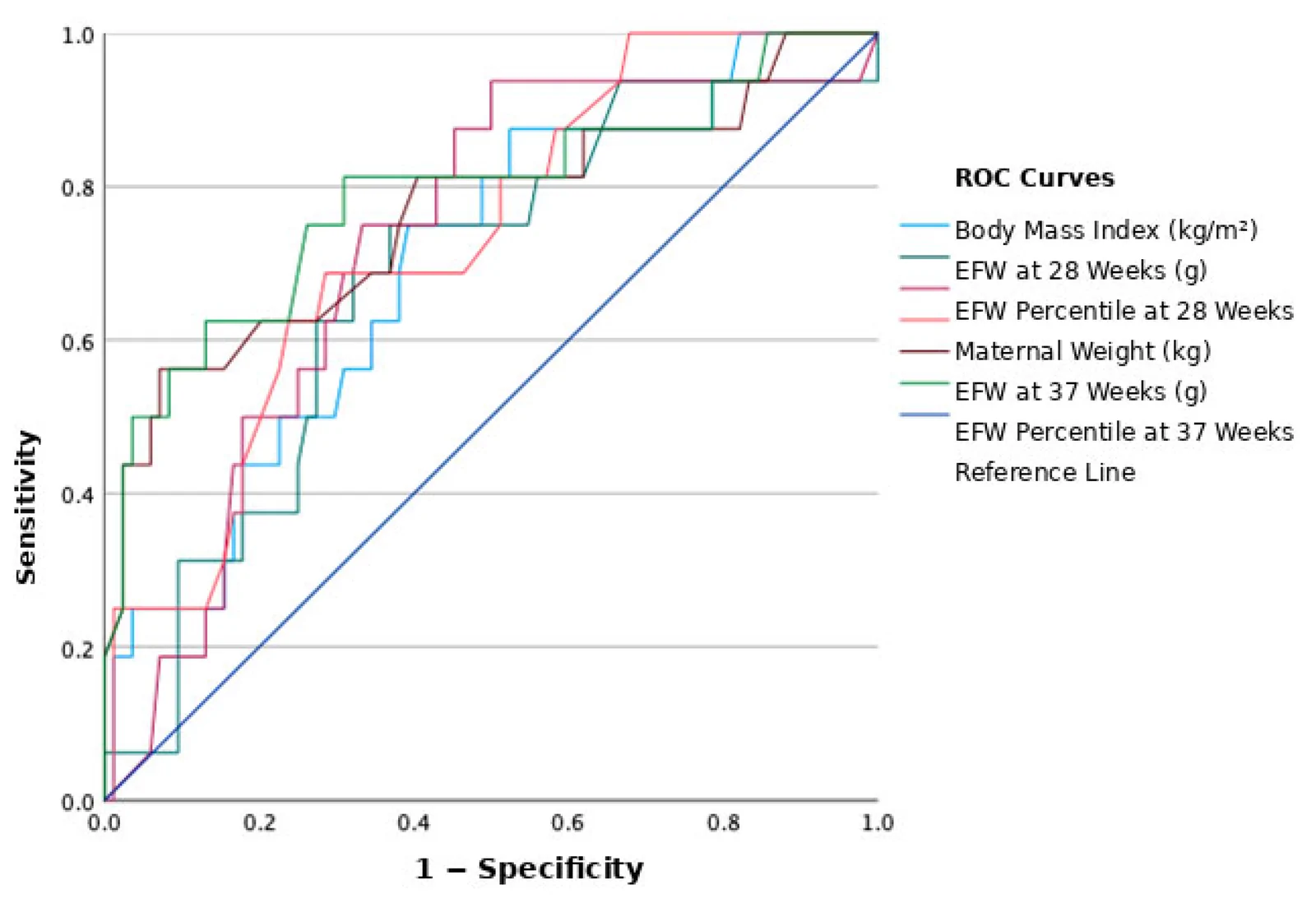
Receiver operating characteristic (ROC) curves illustrating the diagnostic performance of maternal anthropometric characteristics and fetal biometric parameters for predicting large-for-gestational-age (LGA) neonatal outcomes within an unselected obstetric population.

The diagnostic performance of maternal characteristics and fetal biometric measurements for predicting small-for-gestational-age (SGA) and large-for-gestational-age (LGA) neonatal outcomes is summarized in Table 9, with the corresponding ROC curves presented in Figure 1 and Figure 2. For prediction of SGA, maternal characteristics, 28-week fetal biometric parameters, and cerebroplacental ratio (CPR) demonstrated limited discriminatory performance. In contrast, 37-week estimated fetal weight (EFW) and 37-week EFW percentile showed good discriminatory performance, with AUC values of 0.812 and 0.833, respectively (both *p* < 0.001). Similarly, prediction of LGA was primarily driven by maternal anthropometric characteristics and late-pregnancy fetal biometry. Maternal body mass index, maternal booking weight, 28-week EFW, 28-week EFW percentile, 37-week EFW, and 37-week EFW percentile all demonstrated significant discriminatory ability, with AUC values ranging from 0.679 to 0.789 (*p* < 0.05). Optimized cut-off values, corresponding sensitivities, and specificities are presented in Table 9. Overall, late-pregnancy fetal biometric measurements showed greater discriminatory performance than cerebroplacental ratio for predicting neonatal growth abnormalities (Table 9; Figure 1 and Figure 2).

## 4. Discussion

The present prospective longitudinal study evaluated the relationship between serial fetal biometric measurements, Doppler-derived cerebroplacental ratio (CPR), and perinatal outcomes in an unselected obstetric population undergoing routine surveillance. Several key findings emerged from our parsimonious analyses. First, gestational age-specific CPR thresholds at both 28 and 37 weeks demonstrated limited independent association with clinical obstetric and neonatal outcomes. Second, late-pregnancy fetal biometry—specifically 37-week estimated fetal weight (EFW) and EFW percentile—demonstrated significantly superior discriminatory performance for identifying neonatal growth abnormalities compared to standalone Doppler parameters. Third, multivariable regression models identified 37-week EFW and gestational age at delivery as independent determinants of actual birth weight, whereas neither CPR nor earlier biometric tracks independently predicted NICU admission or composite adverse perinatal outcomes. Finally, our rigorous internal validation via 1000 bootstrap resamples confirmed that after adjusting for small-sample optimism, the standalone predictive performance of third-trimester CPR remained close to chance, confirming its limited incremental utility beyond routine biometry in unselected screening cohorts.

The modest predictive performance of the cerebroplacental ratio (CPR) observed in our study aligns with previous large-scale screenings in unselected populations. In an extensive prospective study of over 47,000 pregnancies, Akolekar et al. [14] demonstrated that while reduced CPR statistically correlates with adverse perinatal events, its standalone screening capacity remains strictly limited, especially in appropriately grown fetuses. Our findings corroborate these insights. Although pregnancies in our cohort with lower gestational age-specific CPR percentiles exhibited expected physiological alterations—such as lower middle cerebral artery and higher umbilical artery pulsatility indices—these hemodynamic adaptations did not translate into significant differences in adverse obstetric or neonatal outcomes. Consequently, CPR measured at either 28 or 37 weeks yielded limited discrimination for composite adverse perinatal outcomes, confirming that isolated CPR screening provides negligible prognostic precision in predominantly healthy fetal cohorts.

The findings of a subsequent prospective screening study further support the limited standalone predictive value of CPR in late pregnancy [15]. Although CPR was significantly associated with birth weight Z-scores and umbilical cord blood pH, its performance for predicting adverse perinatal outcomes remained modest. Improved sensitivity at lower CPR thresholds was achieved only at the expense of substantially higher false-positive rates. These observations are highly consistent with our findings. Regardless of whether gestational age-specific 5th or 10th percentile thresholds were applied, CPR was not independently associated with composite adverse perinatal outcomes, NICU admission, Apgar scores, or umbilical cord blood pH. Collectively, these findings suggest that despite its physiological relevance, CPR should be interpreted as an adjunct to comprehensive fetal assessment rather than as an isolated predictor of adverse neonatal outcomes in unselected obstetric cohorts.

The influence of maternal clinical characteristics on CPR has also been investigated in previous studies. In a descriptive–analytical study including 230 singleton pregnancies between 28 and 38 weeks of gestation, Barati et al. reported significantly lower CPR values among women with diabetes mellitus and gestational hypertension [16]. Conversely, no significant associations were observed with maternal body mass index or pregnancy-associated plasma protein-A. Reduced CPR values were also observed across a wide range of estimated fetal weight percentiles rather than being confined exclusively to growth-restricted fetuses. Although our study was not specifically designed to evaluate maternal clinical risk factors, our findings similarly suggest that CPR alone provides limited discriminatory value for predicting adverse perinatal outcomes in an unselected obstetric population. Variations between studies likely reflect differences in study design, patient selection, and baseline population characteristics.

The physiological foundation of CPR assessment, established by Baschat and Gembruch, demonstrates that circulatory impedance evolves dynamically with advancing gestation [17]. Their landmark work validated the use of gestational age-specific reference curves over static cut-off thresholds. We integrated this physiological concept by applying gestational age-specific percentiles for all subgroup stratifications. However, despite controlling for these physiological variations, CPR exhibited poor discriminatory performance for composite adverse events across all threshold definitions. These results suggest that while CPR effectively reflects fetal hemodynamic adaptation, its prospective utility for predicting clinical morbidity remains limited in unselected obstetric cohorts approaching term.

The limited discriminatory performance of fetal biometry for predicting composite adverse perinatal outcomes observed in our study is supported by the prospective cohort study of Basuki et al. [18]. In their evaluation of 938 singleton pregnancies with serial fetal biometry performed at approximately 32 and 37 weeks of gestation, the authors demonstrated that neither conditional abdominal circumference nor estimated fetal weight growth assessments showed satisfactory diagnostic performance for identifying late fetal growth restriction or small-for-gestational-age neonates. Our findings are broadly consistent with these observations. Although 37-week estimated fetal weight and its corresponding percentile showed good discriminatory performance for predicting small-for-gestational-age outcomes in our cohort, standalone fetal biometry did not demonstrate adequate discriminatory performance for predicting comprehensive composite adverse perinatal outcomes. Collectively, these findings suggest that fetal biometry provides greater value for identifying neonatal growth abnormalities rather than predicting broader adverse perinatal outcomes when used in isolation.

Further evidence supporting the limited predictive value of CPR for adverse perinatal outcomes was provided by Bonnevier et al. [19]. In that cohort, Doppler metrics demonstrated poor discriminatory performance for predicting perinatal asphyxia or mortality, which directly corroborates our findings where neither 28-week nor 37-week CPR independently predicted composite adverse perinatal outcomes, NICU admission, or abnormal umbilical cord blood pH. In contrast, late third-trimester fetal biometric assessment, particularly 37-week estimated fetal weight (EFW) and its corresponding percentile, showed significantly superior performance for identifying neonatal growth abnormalities.

Our findings are further strongly supported by a recent comprehensive systematic review published in this journal by Novillo-Del Álamo et al. [20], which evaluated 16 observational studies and 14,823 pregnancies. The authors found that although a low CPR was associated with an increased risk of emergency cesarean delivery for intrapartum fetal compromise, its standalone predictive performance was generally poor and highly heterogeneous across screening cohorts. Importantly, the authors emphasized that CPR prognostic accuracy substantially deteriorated as the interval between the Doppler assessment and delivery increased. This finding may partly explain the limited predictive performance observed in our cohort, in which the interval between the 37-week surveillance scan and actual delivery averaged 13.3 ± 7.1 days.

Notably, however, our findings also closely parallel the large-scale cohort dynamics reported by Stumpfe et al. [21], who evaluated 3326 term pregnancies, including a robust low-risk subgroup of 3105 cases. In their low-risk cohort, the AUC of CPR was strictly limited, with AUC values of 0.516 for NICU admission and 0.517 for the composite adverse perinatal outcome, closely mirroring the near-chance discrimination observed in our unselected population. Notably, this minimal performance was observed despite a median scan-to-delivery interval of only 1 day in their study, suggesting that the modest predictive capacity of CPR in general screening populations may not be exclusively attributable to the chronological gap between Doppler evaluation and delivery.

Consequently, these findings reinforce that combining serial fetal biometry remains superior for growth abnormality surveillance, whereas cross-sectional term CPR metrics cannot sustain clinical utility as a standalone screening vector. This comprehensive clinical framework aligns with a recent review by Danciu and Simionescu [22], who noted that the prognostic utility of third-trimester Doppler metrics is maximized only when tightly integrated into temporal, trend-based multi-marker surveillance rather than interpreted as an isolated predictor in routine practice.

The superiority of late third-trimester fetal biometry over serial growth trajectories and Doppler indices for predicting growth restriction has been verified extensively. In a large prospective screening of 44,043 singleton pregnancies, Ciobanu et al. demonstrated that estimated fetal weight (EFW) measured at 35–37 weeks successfully identified approximately two-thirds of small-for-gestational-age (SGA) neonates and over three-quarters of severe SGA cases [23]. Notably, sequential longitudinal growth velocity provided limited incremental predictive value beyond this single term evaluation. These findings are highly consistent with our results. Although 37-week EFW and its corresponding percentile showed good standalone discriminatory performance for predicting SGA outcomes in our cohort, neither earlier mid-trimester biometry nor sequential CPR scans substantially improved this detection rate. Collectively, these insights reinforce that fetal biometry performed during the late third trimester provides superior clinical precision for identifying neonatal growth abnormalities compared to longitudinal trajectories or isolated Doppler metrics.

Comparable conclusions were reported by D’Antonio et al., who prospectively evaluated 600 singleton pregnancies undergoing third-trimester Doppler assessment [24]. Although CPR and middle cerebral artery pulsatility index correlated statistically with composite adverse perinatal outcomes, their clinical discriminatory performance remained poor. Consequently, the authors did not support using CPR as a standalone screening test at term. Our findings completely mirror these observations. In the present study, CPR measured at either 28 or 37 weeks demonstrated limited independent performance for predicting composite adverse perinatal outcomes. Conversely, late third-trimester fetal biometric assessment, particularly 37-week estimated fetal weight and its corresponding percentile, showed significantly superior discriminatory performance for identifying neonatal growth abnormalities. Collectively, these findings reinforce the prospective value of late third-trimester fetal biometry while supporting the clinical role of CPR as a complementary rather than standalone component of comprehensive fetal assessment.

The limited clinical utility of CPR in late pregnancy has also been demonstrated when compared with alternative Doppler-derived indices. In a secondary analysis of a prospective cohort including 600 singleton pregnancies assessed between 36 and 37 weeks of gestation, Di Mascio et al. compared the diagnostic performance of the cerebroplacental ratio (CPR) with the umbilicocerebral ratio (UCR) for predicting composite adverse perinatal outcomes [25]. Although pregnancies with adverse outcomes exhibited lower CPR and higher UCR values, the discriminatory performance of both indices remained poor, with no significant difference between the two ratios. These findings are highly consistent with the results of the present study. Regardless of whether gestational age-specific 5th or 10th percentile thresholds were applied, CPR demonstrated limited discriminatory performance for predicting adverse perinatal outcomes. Collectively, these findings suggest that alternative formulations of Doppler-derived indices provide little additional clinical value over CPR for routine third-trimester fetal surveillance.

Conflicting findings have occasionally been reported in smaller observational studies. Elraey et al. described an association between low CPR values and adverse neonatal outcomes in a prospective study of 40 uncomplicated singleton pregnancies [26]. However, the small sample size, use of a fixed CPR cut-off, and limited statistical power restrict the generalizability of those findings. In contrast, the individual participant data meta-analysis by Heidweiller-Schreurs et al., including 18,731 singleton pregnancies from 17 datasets, demonstrated that although CPR was significantly associated with adverse perinatal outcomes, its addition to umbilical artery Doppler provided only minimal improvement in discriminatory performance [27]. These high-level data are entirely consistent with our results. Despite using gestational age-specific CPR percentile thresholds, neither 28-week nor 37-week CPR independently predicted adverse perinatal outcomes in our cohort. Collectively, these findings indicate that although CPR reflects fetal hemodynamic adaptation, its incremental value beyond conventional fetal biometry remains limited in unselected obstetric cohorts approaching term.

The findings reported by Kahramanoğlu et al. further suggest that the clinical value of CPR depends largely on the specific population being studied [28]. In their retrospective cohort of 407 pregnancies complicated by late-onset fetal growth restriction, a CPR below the 5th percentile was independently associated with adverse perinatal outcomes, including lower birth weight, earlier delivery, and increased NICU admissions. These results differ from our prospective outcomes, where neither 28-week nor 37-week CPR independently predicted adverse neonatal events. This discrepancy is directly attributable to differences in the baseline study populations. Whereas Kahramanoğlu et al. exclusively evaluated high-risk pregnancies affected by late-onset growth restriction, our cohort represented an unselected obstetric population with predominantly appropriately grown fetuses. Collectively, these insights demonstrate that while CPR maintains substantial clinical utility in pregnancies with established placental insufficiency, its performance fails as a universal screening tool for the general unselected obstetric population.

This interpretation is further supported by the review by Kalafat and Khalil and the retrospective study by Mecke et al. [29,30]. Kalafat and Khalil emphasized that although reduced CPR reflects fetal adaptive responses to placental insufficiency, its discriminatory performance is substantially greater in suspected small-for-gestational-age or late-onset fetal growth restriction than in appropriately grown fetuses. Similarly, Mecke et al. demonstrated that among appropriately grown fetuses, reduced CPR was associated with an increased risk of intrapartum fetal distress and secondary cesarean delivery, but showed limited associations with objective neonatal outcomes such as Apgar scores and umbilical artery pH. These findings are consistent with our results. Although CPR may identify subtle fetal hemodynamic adaptation in selected pregnancies, it did not provide meaningful additional prognostic information for composite adverse perinatal outcomes in our unselected cohort. In contrast, late third-trimester fetal biometric assessment, particularly 37-week estimated fetal weight and its corresponding percentile, provided significantly greater discriminatory performance for identifying neonatal growth abnormalities.

More recent evidence also supports the concept that refinement of fetal growth definitions or Doppler-derived indices provides only limited improvement in predicting neonatal morbidity. In the secondary analysis of the NICHD Fetal Growth Studies, Shea et al. demonstrated that expanding the definition of fetal growth restriction by incorporating abdominal circumference below the 10th percentile increased the number of pregnancies classified as growth restricted but did not improve prediction of composite neonatal morbidity [31]. Similarly, Sirico et al. showed that adjusting CPR for estimated fetal weight strengthened statistical associations but did not result in clinically meaningful improvements in screening performance [32]. Furthermore, Yagel et al. highlighted several methodological limitations that continue to restrict the routine clinical application of CPR, including gestational age-dependent physiological variation, heterogeneous cut-off definitions, measurement variability, and the limited balance between sensitivity and specificity in unselected populations [33]. These findings are consistent with our results. Despite evaluating CPR using gestational age-specific percentile thresholds at two different time points during the third trimester, its incremental value for predicting adverse perinatal outcomes remained limited. Collectively, the available evidence suggests that although CPR provides valuable physiological information and may have a role in selected high-risk pregnancies, routine third-trimester surveillance in unselected screening cohorts continues to rely primarily on conventional fetal biometric assessment.

One of the major strengths of the present study is its prospective longitudinal design, which enabled serial assessment of fetal biometry and Doppler parameters within the same pregnancies at both 28 and 37 weeks of gestation. Unlike cross-sectional studies, this approach allowed evaluation of temporal changes in fetal growth and hemodynamic adaptation during the third trimester. Another important strength is that all ultrasonographic and Doppler examinations were performed by a single experienced operator using a standardized protocol, thereby minimizing interobserver variability. In addition, the simultaneous evaluation of fetal biometric measurements and Doppler-derived parameters within the same prospective cohort allowed direct comparison of their relative discriminatory performance. Furthermore, maternal characteristics, fetal biometric measurements, Doppler indices, delivery data, and neonatal outcomes were collected prospectively, providing a comprehensive assessment of the relationship between antenatal findings and perinatal outcomes in a well-characterized unselected obstetric population.

Several limitations should also be acknowledged. First, this was a single-center study with a modest sample size, which was determined by the number of consecutive eligible participants during a predefined recruitment period rather than a formal a priori power analysis. Despite the parsimonious structure of our revised models, the limited number of NICU (*n* = 16) and adverse outcome (*n* = 26) events reduced statistical precision and may have resulted in residual small-sample bias. Second, our cohort represented a localized unselected obstetric population with a high prevalence of repeat cesarean sections (37%), reflecting regional healthcare dynamics in Türkiye. This specific demographic context may limit the direct generalizability of our findings to strictly low-risk universal screening cohorts. Third, because our longitudinal protocol required completion of both the 28-week and 37-week scans, individuals who delivered prematurely before the second scheduled examination were inherently excluded from the final analysis, which constitutes a potential selection bias and limits the applicability of our findings to early late-onset growth restriction dynamics. Fourth, although serial Doppler examinations were performed at two standardized gestational ages, additional assessments closer to delivery might have provided further information regarding acute fetal deterioration. This factor is clinically relevant given that the interval between our 37-week surveillance scan and actual delivery averaged 13.3 ± 7.1 days. Consequently, external validation in larger multicenter prospective longitudinal cohorts remains an absolute prerequisite before routine clinical implementation.

In conclusion, the findings of the present study suggest that routine third-trimester fetal biometry, particularly estimated fetal weight assessed close to delivery, remains the most informative parameter for identifying neonatal growth abnormalities in an unselected obstetric population. In contrast, although the cerebroplacental ratio reflects fetal circulatory adaptation to placental insufficiency, its isolated use provided limited additional prognostic information for predicting broader composite adverse perinatal outcomes or adverse neonatal outcomes. Therefore, CPR should be interpreted as a complementary Doppler parameter within a comprehensive multi-marker fetal assessment framework rather than as a standalone screening tool in routine clinical practice.

## 5. Conclusions

In this prospective longitudinal study of an unselected obstetric population, serial third-trimester assessment of the cerebroplacental ratio (CPR) provided limited additional value for predicting adverse perinatal outcomes. Regardless of whether gestational age-specific 5th or 10th percentile thresholds were applied, parsimonious regression models demonstrated that CPR did not independently predict adverse perinatal outcomes, NICU admission, low Apgar scores, or abnormal umbilical cord blood pH.

In contrast, late third-trimester fetal biometric assessment, particularly estimated fetal weight (EFW) and EFW percentile measured at 37 weeks of gestation, demonstrated significantly superior discriminatory performance for identifying neonatal growth abnormalities, including small- and large-for-gestational-age infants. However, even standalone fetal biometry showed limited capacity to predict complex neonatal morbidity, emphasizing the multifactorial nature of adverse perinatal outcomes.

These findings suggest that routine third-trimester fetal surveillance in unselected screening cohorts should continue to rely primarily on conventional fetal biometric assessment. Conversely, CPR should be interpreted as a complementary Doppler parameter within a comprehensive multi-marker framework rather than as a standalone screening tool. Future large-scale, multicenter prospective longitudinal studies are warranted to better define the specific clinical scenarios in which serial Doppler metrics may provide meaningful additional prognostic resolution.

## Figures and Tables

**Table 1:** Baseline Maternal Demographic, Anthropometric, and Obstetric Characteristics of the Study Population (*n* = 100).

Parameter	Value (Mean ± SD) or *n* (%)
Maternal Age (years)	29.2 ± 4.7
Maternal Height (cm)	162.1 ± 6.4
Maternal Weight (kg)	81.5 ± 16.2
Body Mass Index (BMI, kg/m^2^)	31.1 ± 6.3
Gravidity	
1 (Primigravida)	37 (37.0%)
2	35 (35.0%)
3	18 (18.0%)
≥4	10 (10.0%)
Parity	
0 (Nulliparous)	45 (45.0%)
1	36 (36.0%)
2	15 (15.0%)
≥3	4 (4.0%)
Living Children	
0	45 (45.0%)
1	39 (39.0%)
2	12 (12.0%)
≥3	4 (4.0%)
History of Abortion	
0	77 (77.0%)
1	15 (15.0%)
2	6 (6.0%)
≥3	2 (2.0%)

Note: Continuous variables are presented as mean ± standard deviation (SD), whereas categorical variables are presented as number (percentage). BMI, body mass index.

**Table 2:** Comparative Analysis of Fetal Biometric Indices, Maternal–Fetal Doppler Velocimetry, and Growth Classifications Between 28 Weeks and 37 Weeks of Gestation (*n* = 100).

Parameters	28 Weeks of Gestation (Mean ± SD) or *n* (%)	37 Weeks of Gestation (Mean ± SD) or *n* (%)
Biometric Gestational Age Estimates (days)		
Biparietal Diameter (BPD)	204.1 ± 10.8	258.5 ± 11.7
Head Circumference (HC)	205.2 ± 10.7	257.6 ± 13.1
Abdominal Circumference (AC)	201.2 ± 10.3	258.7 ± 14.6
Femur Length (FL)	200.0 ± 9.4	257.3 ± 13.3
Fetal Weight Estimates		
Estimated Fetal Weight (EFW, g)	1317.3 ± 306.9	3048.4 ± 447.7
EFW Percentile	67.1 ± 33.0	61.1 ± 28.7
Doppler Impedance Indices		
MCA Pulsatility Index (PI)	2.4 ± 0.7	2.4 ± 0.6
MCA Resistance Index (RI)	1.0 ± 0.1	1.0 ± 0.2
UA Pulsatility Index (PI)	1.2 ± 0.3	1.1 ± 0.3
UA Resistance Index (RI)	0.7 ± 0.1	0.7 ± 0.1
Cerebroplacental Ratio (CPR)	2.1 ± 0.7	1.8 ± 0.5
Growth and Fluid Categories, *n* (%)		
EFW Percentile Groups		
<10th percentile	8 (8.0%)	5 (5.0%)
10th–90th percentile	52 (52.0%)	79 (79.0%)
>90th percentile	40 (40.0%)	16 (16.0%)
Amniotic Fluid Index (AFI)		
Normal/Adequate	97 (97.0%)	94 (94.0%)
Oligohydramnios	0 (0.0%)	3 (3.0%)
Polyhydramnios	3 (3.0%)	3 (3.0%)
CPR Percentile Classifications		
<5th percentile	21 (21.0%)	32 (32.0%)
≥5th percentile	79 (79.0%)	68 (68.0%)

Note: Data are presented as mean ± standard deviation (SD) for continuous variables and as number (percentage) for categorical variables. Abbreviations: BPD, biparietal diameter; HC, head circumference; AC, abdominal circumference; FL, femur length; EFW, estimated fetal weight; MCA, middle cerebral artery; UA, umbilical artery; PI, pulsatility index; RI, resistance index; CPR, cerebroplacental ratio; AFI, amniotic fluid index.

**Table 3:** Delivery Characteristics, Intrapartum Indications, and Neonatal Clinical Status (*n* = 100).

Parameter	Value (Mean ± SD) or *n* (%)
Delivery Characteristics	
Interval from 37-Week Examination to Delivery (days)	13.3 ± 7.1
Gestational Age at Birth (weeks)	38.6 ± 1.0
Mode of Delivery	
Spontaneous Vaginal Delivery (SVD)	38 (38.0%)
Cesarean Section (C/S)	62 (62.0%)
Indications for Cesarean Section (*n* = 62)	
Previous Cesarean Section	37 (37.0%)
Cephalopelvic Disproportion (CPD)	15 (15.0%)
Acute Fetal Distress	5 (5.0%)
Preeclampsia	3 (3.0%)
Breech Presentation	1 (1.0%)
Fetal Omphalocele	1 (1.0%)
Neonatal Anthropometrics	
Birth Weight (g)	3252.5 ± 520.8
Birth Length (cm)	49.9 ± 2.5
Birth Weight Percentile Category	
<10th percentile (SGA)	12 (12.0%)
10th–90th percentile (AGA)	72 (72.0%)
>90th percentile (LGA)	16 (16.0%)
Cord Blood Gas Analysis	
Umbilical Cord pH	7.3 ± 0.1
pO_2_ (mmHg)	38.9 ± 28.3
pCO_2_ (mmHg)	42.5 ± 7.7
Apgar Scores	
1 min Apgar < 7	6 (6.0%)
1 min Apgar ≥ 7	94 (94.0%)
5 min Apgar < 7	1 (1.0%)
5 min Apgar ≥ 7	99 (99.0%)
NICU Outcomes	
NICU Admission Required	16 (16.0%)
NICU Length of Stay (days)	0.9 ± 2.3
Composite Adverse Perinatal Outcome	26 (26.0%)

Note: Continuous variables are presented as mean ± standard deviation (SD), whereas categorical variables are presented as number (percentage). Abbreviations: SGA, small for gestational age; AGA, appropriate for gestational age; LGA, large for gestational age; NICU, neonatal intensive care unit.

**Table 4:** Comparative Analysis of Maternal Demographics, Fetal Growth, and Obstetric Outcomes Stratified by 28-Week Cerebroplacental Ratio (CPR) 5th Percentile Classification (*n* = 100).

Clinical Parameter	CPR < 5th Percentile (*n* = 21)	CPR ≥ 5th Percentile (*n* = 79)	*p*-Value
Maternal Age (years)	30.2 ± 4.8	28.9 ± 4.7	0.423
Body Mass Index (BMI, kg/m^2^)	29.5 ± 6.6	31.5 ± 6.2	0.198
28-Week EFW Percentile	69.4 ± 36.0	66.5 ± 32.4	0.506
28-Week EFW Group, *n* (%)			0.353
<10th percentile	2 (9.5%)	6 (7.6%)	
10th–90th percentile	9 (42.9%)	43 (54.4%)	
>90th percentile	10 (47.6%)	30 (38.0%)	
37-Week EFW Percentile	58.7 ± 29.2	61.7 ± 28.8	0.609
37-Week EFW Group, *n* (%)			0.280
<10th percentile	2 (9.5%)	3 (3.8%)	
10th–90th percentile	17 (81.0%)	62 (78.5%)	
>90th percentile	2 (9.5%)	14 (17.7%)	
Gestational Age at Birth (weeks)	38.8 ± 1.0	38.6 ± 1.0	0.399
Exam-to-Delivery Interval (days)	14.2 ± 6.6	13.1 ± 7.2	0.689
Cesarean Section Indications, *n* (%)			0.466
Previous Cesarean Section	7 (18.9%)	30 (81.1%)	
Cephalopelvic Disproportion	2 (13.3)	13 (86.7%)	
Acute Fetal Distress	1 (20%)	4 (80%)	
Preeclampsia	1 (33.3%)	2 (66.7%)	
Breech Presentation/Structural Omphalocele	1 (4.8%)	1 (1.3%)	
Neonatal Birth Weight (g)	3117.9 ± 421.6	3288.3 ± 540.9	0.104
Birth Weight Category, *n* (%)			0.645
<10th percentile (SGA)	4 (19.0%)	8 (10.1%)	
10th–90th percentile (AGA)	15 (71.4%)	57 (72.2%)	
>90th percentile (LGA)	2 (9.5%)	14 (17.7%)	
Neonatal Sex Allocation, *n* (%)			0.474
Female	8 (38.1%)	37 (46.8%)	
Male	13 (61.9%)	42 (53.2%)	
Composite Adverse Outcome, *n* (%)			0.797
Absent	16 (76.2%)	58 (73.4%)	
Present	5 (23.8%)	21 (26.6%)	

Note: Continuous variables are presented as mean ± standard deviation (SD), whereas categorical variables are presented as number (percentage). EFW, estimated fetal weight; CPR, cerebroplacental ratio; SGA, small for gestational age; AGA, appropriate for gestational age; LGA, large for gestational age.

**Table 5:** Comprehensive Longitudinal Analysis of Fetal Biometry, Maternal–Fetal Doppler Velocimetry, and Neonatal Outcomes Stratified by 28-Week and 37-Week Cerebroplacental Ratio (CPR) 10th Percentile Thresholds (*n* = 100).

Clinical Parameter	28-Week CPR < 10th Percentile (*n* = 25)	28-Week CPR ≥ 10th Percentile (*n* = 75)	*p*-Value	37-Week CPR < 10th Percentile (*n* = 44)	37-Week CPR ≥ 10th Percentile (*n* = 56)	*p*-Value
Maternal Age (years)	30.1 ± 4.7	28.9 ± 4.7	0.365	29.3 ± 5.1	29.1 ± 4.5	0.956
Body Mass Index (BMI, kg/m^2^)	29.5 ± 6.1	31.6 ± 6.3	0.148	29.7 ± 5.4	32.2 ± 6.8	0.101
28-Week Gestational Age (days)	199.3 ± 2.6	199.6 ± 2.6	0.579	199.5 ± 2.4	199.5 ± 2.7	0.867
28-Week BPD (days)	204.5 ± 11.6	204.0 ± 10.6	0.756	204.3 ± 9.4	204.0 ± 12.0	0.692
28-Week HC (days)	205.4 ± 13.0	205.1 ± 9.9	1.000	205.0 ± 9.2	205.4 ± 11.8	0.917
28-Week AC (days)	197.8 ± 10.6	202.4 ± 10.0	0.092	198.7 ± 9.5	203.2 ± 10.5	0.032
28-Week FL (days)	197.7 ± 10.7	200.8 ± 8.9	0.321	199.0 ± 10.1	200.9 ± 8.8	0.600
28-Week EFW (g)	1291.0 ± 311.5	1326.1 ± 307.0	0.842	1272.6 ± 264.1	1352.5 ± 334.9	0.304
28-Week EFW Percentile	64.7 ± 38.1	67.9 ± 31.4	0.883	63.1 ± 35.8	70.2 ± 30.7	0.198
28-Week MCA PI	1.9 ± 0.5	2.6 ± 0.7	<0.001	2.3 ± 0.6	2.4 ± 0.8	0.657
28-Week MCA RI	0.9 ± 0.1	1.0 ± 0.1	0.012	1.0 ± 0.1	1.0 ± 0.2	0.209
28-Week UA PI	1.4 ± 0.4	1.1 ± 0.2	<0.001	1.3 ± 0.4	1.1 ± 0.2	0.001
28-Week UA RI	0.7 ± 0.2	0.7 ± 0.1	0.403	0.7 ± 0.1	0.7 ± 0.1	0.158
28-Week Absolute CPR	—	—	—	1.8 ± 0.6	2.3 ± 0.7	0.001
Exam-to-Delivery Interval (days)	14.2 ± 6.1	13.0 ± 7.4	0.609	13.1 ± 6.9	13.4 ± 7.2	0.565
Umbilical Cord Blood pH	7.3 ± 0.1	7.3 ± 0.1	0.962	7.3 ± 0.1	7.3 ± 0.1	0.876
Umbilical Cord pO_2_ (mmHg)	—	—	—	40.5 ± 20.3	37.6 ± 33.4	0.047
Umbilical Cord pCO_2_ (mmHg)	—	—	—	42.3 ± 7.3	42.7 ± 8.1	0.702
NICU Length of Stay (days)	1.1 ± 2.4	0.8 ± 2.3	0.558	1.1 ± 2.6	0.8 ± 2.0	0.542
Neonatal Birth Weight (g)	3117.6 ± 404.9	3297.5 ± 549.1	0.070	3216.3 ± 440.9	3280.9 ± 578.3	0.437
Neonatal Birth Length (cm)	49.2 ± 2.3	50.1 ± 2.5	0.087	49.3 ± 2.3	50.4 ± 2.5	0.028
Gestational Age at Birth (weeks)	38.8 ± 0.9	38.6 ± 1.0	0.327	38.7 ± 1.1	38.6 ± 1.0	0.517

Note: Data are presented as mean ± standard deviation (SD). BPD, biparietal diameter; HC, head circumference; AC, abdominal circumference; FL, femur length; EFW, estimated fetal weight; MCA, middle cerebral artery; UA, umbilical artery; PI, pulsatility index; RI, resistance index; CPR, cerebroplacental ratio; NICU, neonatal intensive care unit.

**Table 6:** Maternal Characteristics, Fetal Growth Categories, and Perinatal Outcomes According to the 37-Week Cerebroplacental Ratio (CPR) 10th Percentile Threshold (*n* = 100).

Clinical Parameter	37-Week CPR < 10th Percentile (*n* = 44)	37-Week CPR ≥ 10th Percentile (*n* = 56)	*p*-Value
Gravidity, *n* (%)			0.550
1 (Primigravida)	21 (47.7%)	16 (28.6%)	
≥2	23 (52.3%)	40 (71.4%)	
Parity, *n* (%)			0.090
0 (Nulliparous)	24 (54.5%)	21 (37.5%)	
≥1	20 (45.5%)	35 (62.5%)	
History of Abortion, *n* (%)			0.954
Absent	34 (77.3%)	43 (76.8%)	
Present (≥1 spontaneous abortion)	10 (22.7%)	13 (23.2%)	
28-Week EFW Percentile Group, *n* (%)			0.149
<10th percentile	6 (13.6%)	2 (3.6%)	
10th–90th percentile	23 (52.3%)	29 (51.8%)	
>90th percentile	15 (34.1%)	25 (44.6%)	
37-Week EFW Percentile Group, *n* (%)			0.035
<10th percentile	1 (2.3%)	4 (7.1%)	
10th–90th percentile	40 (90.9%)	39 (69.6%)	
>90th percentile	3 (6.8%)	13 (23.2%)	
Mode of Delivery			0.595
Spontaneous Vaginal Delivery (SVD)	18 (40.9%)	20 (35.7%)	
Cesarean Section (C/S)	26 (59.1%)	36 (64.3%)	
Indications for Cesarean Section (*n* = 62), *n* (%)			0.074
Previous Cesarean Section	18 (69.3%)	19 (52.8%)	
Cephalopelvic Disproportion (CPD)	3 (11.6%)	12 (33.4%)	
Acute Fetal Distress	2 (7.7%)	3 (8.3%)	
Preeclampsia	1 (3.8%)	2 (5.5%)	
Breech Presentation/Fetal Omphalocele	2 (7.6%)	0 (0.0%)	
1 min Apgar Score, *n* (%)			0.321
<7	3 (6.9%)	3 (5.4%)	
≥7	41 (93.1%)	53 (94.6%)	
5 min Apgar Score, *n* (%)			0.506
<7	1 (2.3%)	0 (0.0%)	
≥7	43 (97.7%)	56 (100.0%)	
Umbilical Cord Blood pH Category, *n* (%)			0.805
<7.20 (Acidemia)	2 (4.5%)	2 (3.6%)	
≥7.20	42 (95.5%)	54 (96.4%)	
NICU Admission			0.598
Not Required	36 (81.8%)	48 (85.7%)	
Required	8 (18.2%)	8 (14.3%)	
Neonatal Birth Weight Percentile Category (INTERGROWTH-21st)			0.139
<10th percentile (SGA)	4 (9.1%)	8 (14.3%)	
10th–90th percentile (AGA)	36 (81.8%)	36 (64.3%)	
>90th percentile (LGA)	4 (9.1%)	12 (21.4%)	
Composite Adverse Perinatal Outcome, *n* (%)			0.508
Absent	34 (77.3%)	40 (71.4%)	
Present	10 (22.7%)	16 (28.6%)	

Note: Data are presented as number (percentage). EFW, estimated fetal weight; CPR, cerebroplacental ratio; SGA, small for gestational age; AGA, appropriate for gestational age; LGA, large for gestational age; NICU, neonatal intensive care unit; CPD, cephalopelvic disproportion. Percentages for cesarean section indications were calculated among women who underwent cesarean delivery (*n* = 62).

**Table 7:** Parsimonious multivariable regression analyses of factors associated with neonatal outcomes.

Outcome	Model	Predictor	Effect Measure	Estimate	95% CI	*p*-Value
Neonatal birth weight (g)	28-week model	EFW, per 100-g increase	B coefficient	27.771	−4.069 to 59.611	0.087
		CPR, per 0.1-unit increase	B coefficient	15.979	2.194 to 29.763	0.024
		Gestational age at birth, per week	B coefficient	156.669	60.719 to 252.619	0.002
Neonatal birth weight (g)	37-week model	EFW, per 100-g increase	B coefficient	71.593	54.012 to 89.174	<0.001
		CPR, per 0.1-unit increase	B coefficient	8.434	−6.038 to 22.906	0.250
		Gestational age at birth, per week	B coefficient	85.185	6.795 to 163.576	0.034
NICU admission	28-week model	EFW percentile, per 10-point increase	Adjusted OR	0.952	0.813 to 1.116	0.545
		CPR, per 0.1-unit increase	Adjusted OR	1.027	0.956 to 1.103	0.473
NICU admission	37-week model	EFW percentile, per 10-point increase	Adjusted OR	1.001	0.829 to 1.209	0.994
		CPR, per 0.1-unit increase	Adjusted OR	0.951	0.849 to 1.065	0.383
Composite adverse perinatal outcome	28-week model	EFW percentile, per 10-point increase	Adjusted OR	1.014	0.884 to 1.164	0.840
		CPR, per 0.1-unit increase	Adjusted OR	1.016	0.955 to 1.081	0.612
Composite adverse perinatal outcome	37-week model	EFW percentile, per 10-point increase	Adjusted OR	0.920	0.789 to 1.073	0.290
		CPR, per 0.1-unit increase	Adjusted OR	0.993	0.911 to 1.081	0.865

Linear regression coefficients represent the change in neonatal birth weight in grams. Logistic regression results are presented as adjusted odds ratios. To reduce overfitting and multicollinearity, 28- and 37-week measurements were modeled separately, and EFW percentile obtained at the same examination were not entered simultaneously. NICU and composite-outcome models were restricted a priori to EFW percentile and CPR. The NICU length-of-stay regression was removed because 84 neonates had zero length-of-stay regression and only 16 neonates were admitted.

**Table 8:** Diagnostic Performance of Longitudinal Fetal Biometric Measurements and Maternal–Fetal Doppler Indices in Predicting Composite Adverse Perinatal Outcomes (*n* = 100).

Operational Checkpoint and Test Predictors	Area Under the Curve (AUC)	*p*-Value	95% Confidence Interval (CI)
28 Weeks of Gestation			
Estimated Fetal Weight (EFW, g)	0.552	0.432	0.421–0.683
EFW Gestational Percentile	0.513	0.841	0.381–0.646
MCA Pulsatility Index (PI)	0.533	0.618	0.405–0.661
MCA Resistance Index (RI)	0.520	0.762	0.383–0.657
UA Pulsatility Index (PI)	0.431	0.300	0.307–0.555
UA Resistance Index (RI)	0.399	0.126	0.266–0.532
Cerebroplacental Ratio (CPR)	0.552	0.430	0.425–0.679
37 Weeks of Gestation			
Estimated Fetal Weight (EFW, g)	0.455	0.499	0.312–0.599
EFW Gestational Percentile	0.465	0.599	0.321–0.609
MCA Pulsatility Index (PI)	0.549	0.458	0.416–0.682
MCA Resistance Index (RI)	0.566	0.316	0.444–0.688
UA Pulsatility Index (PI)	0.541	0.540	0.410–0.671
UA Resistance Index (RI)	0.453	0.477	0.325–0.581
Cerebroplacental Ratio (CPR)	0.482	0.786	0.357–0.607

Note: Receiver operating characteristic (ROC) curve analysis was performed to evaluate the ability of fetal biometric parameters and Doppler velocimetry indices to predict composite adverse perinatal outcomes. AUC, area under the curve; CI, confidence interval; EFW, estimated fetal weight; MCA, middle cerebral artery; UA, umbilical artery; PI, pulsatility index; RI, resistance index; CPR, cerebroplacental ratio.

**Table 9:** Diagnostic Performance and Optimized Cutoff Thresholds of Maternal Characteristics and Fetal Biometry in Predicting Small-for-Gestational-Age (SGA) and Large-for-Gestational-Age (LGA) Neonatal Outcomes (*n* = 100).

Evaluated Models and Test Predictor Variables	Area Under the Curve (AUC)	Optimized Cutoff	Sensitivity (%)	Specificity (%)	*p*-Value	95% Confidence Interval (CI)
Model 1: Predicting SGA (<10th Percentile)						
Maternal Age (years)	0.564	—	—	—	0.474	0.394–0.734
Body Mass Index (BMI, kg/m^2^)	0.645	—	—	—	0.103	0.473–0.818
28-Week Estimated Fetal Weight (g)	0.545	—	—	—	0.611	0.384–0.707
28-Week EFW Gestational Percentile	0.560	—	—	—	0.504	0.392–0.727
28-Week Cerebroplacental Ratio (CPR)	0.568	—	—	—	0.445	0.400–0.736
37-Week Estimated Fetal Weight (g)	0.812	—	—	—	<0.001	0.701–0.922
37-Week EFW Gestational Percentile	0.833	—	—	—	<0.001	0.732–0.933
37-Week Cerebroplacental Ratio (CPR)	0.511	—	—	—	0.903	0.355–0.667
Exam-to-Delivery Interval (days)	0.622	—	—	—	0.173	0.442–0.802
Model 2: Predicting LGA (>90th Percentile)						
Maternal Body Mass Index (kg/m^2^)	0.698	31.9	63	70	0.012	0.563–0.834
Maternal Booking Weight (kg)	0.730	85.5	69	72	0.004	0.604–0.855
28-Week Estimated Fetal Weight (g)	0.679	1403.4	63	72	0.024	0.537–0.820
28-Week EFW Gestational Percentile	0.720	90.1	75	67	0.005	0.591–0.849
37-Week Estimated Fetal Weight (g)	0.763	3091.5	81	60	0.001	0.615–0.912
37-Week EFW Gestational Percentile	0.789	87.1	63	87	<0.001	0.648–0.930

Note: Receiver operating characteristic (ROC) curve analysis was used to evaluate the predictive performance of maternal characteristics and fetal biometric measurements for small-for-gestational-age (SGA) and large-for-gestational-age (LGA) neonatal outcomes. Optimized cutoffs were determined using the Youden index. AUC, area under the curve; CI, confidence interval; EFW, estimated fetal weight; CPR, cerebroplacental ratio; BMI, body mass index.

## Data Availability

The datasets generated and/or analyzed during the current study are available from the corresponding author upon reasonable request.
